# An Unusual Cause of Post-cesarean Intraperitoneal Hemorrhage: Inadvertent Intra-abdominal Placement of a Femoral Venous Catheter

**DOI:** 10.7759/cureus.108126

**Published:** 2026-05-02

**Authors:** Mohamed Amine Kamar, Ayoub Chaar

**Affiliations:** 1 Anesthesiology and Critical Care, Centre Hospitalier Universitaire Ibn Sina, Rabat, MAR; 2 Anesthesiology and Critical Care, Centre Hospitalier Provincial Azzamouri, Kenitra, MAR

**Keywords:** catheter malposition, central venous catheterization, femoral vein, hemoperitoneum, obstetric critical care, ultrasound guidance

## Abstract

Severe abruptio placentae is a major obstetric emergency that may require aggressive resuscitation and central venous catheterization. Femoral venous access is often selected in this context because of its accessibility and absence of pneumothorax risk; however, inadvertent intraperitoneal malposition of the catheter is an exceptionally rare and potentially life-threatening complication. We report the case of a 25-year-old woman at 36 weeks of gestation, admitted for abruptio placentae with intrauterine fetal death and hemorrhagic shock, who underwent emergency cesarean section and was transferred to the intensive care unit (ICU) postoperatively. A right femoral central venous catheter was inserted using the landmark-based technique in a single, apparently uneventful attempt. Postoperative anemia prompted transfusion, but hemoglobin paradoxically failed to improve and continued to decline from 8.2 to 7.8 g/dL despite two units of packed red blood cells, with no identifiable external source of bleeding. Progressive pelvic effusion on serial ultrasound, combined with abdominal distension and tachycardia, prompted surgical re-exploration on postoperative day two. Exploration revealed approximately 600 mL of hemoperitoneum, with the catheter tip identified within the right pelvic peritoneal cavity. The catheter was withdrawn under direct visual control with immediate cessation of bleeding, and the patient recovered fully. This case highlights a rare but serious complication of femoral catheterization, underscores the diagnostic importance of paradoxical hemoglobin kinetics as a signal of occult intraperitoneal hemorrhage, and supports systematic ultrasound guidance and post-insertion catheter verification in obstetric critical care settings.

## Introduction

Abruptio placentae is a major obstetric emergency frequently requiring aggressive resuscitation and rapid central venous access. The femoral vein is often selected in this context because of its accessibility, predictable anatomy, and absence of pneumothorax risk, and is conventionally cannulated using the landmark-based technique. While femoral catheterization carries well-recognized complications, malposition of the catheter tip into the peritoneal cavity is an exceptionally rare event, with only a handful of cases reported in the literature [1,2].

We report what appears to be an exceptionally rare case of intraperitoneal femoral catheter malposition occurring in the specific context of post-cesarean care, presenting insidiously as a paradoxical failure of transfusion therapy and diagnosed only during surgical exploration. This case highlights a rare yet life-threatening complication and supports the adoption of systematic ultrasound-guided catheterization and post-insertion catheter verification in obstetric critical care settings.

Written informed consent was obtained from the patient for publication of this case report and accompanying intraoperative images.

## Case presentation

A 25-year-old parturient with no significant medical, surgical, or allergic history, gravida 3 para 3 with 2 living children (G3P3L2), at 36 weeks and two days of gestation, had been previously followed at a primary birthing center. She was initially admitted to that facility for threatened preterm labor, with stable vital signs, blood pressure (BP) 130/80 mmHg, and heart rate (HR) 70 bpm, and was placed on oxygen therapy before being transferred to a level-2 maternity hospital.

Upon arrival, the patient presented with profuse vaginal bleeding and hemorrhagic shock, with vital signs revealing severe hemodynamic compromise, BP 80/50 mmHg, and HR 140 bpm. Obstetric examination revealed absent fetal heart tones, and bedside ultrasound confirmed the diagnosis of retroplacental hematoma (abruptio placentae).

Simultaneous resuscitation measures were initiated, including placement of two large-bore peripheral intravenous lines, volume resuscitation, urinary catheterization, and administration of a loading dose of tranexamic acid. Blood samples were drawn, and following initial resuscitation, the patient was hemodynamically stabilized and transferred to the operating room for emergency cesarean section. General anesthesia was induced without incident, with no hemodynamic instability or circulatory collapse noted during induction.

Intraoperative findings included a retroplacental hematoma involving one-third of the placental surface. A fresh stillborn male infant weighing 2800 g was delivered. The uterus was closed without a hemodynamic incident.

Pre-operative laboratory results revealed normocytic anemia with a hemoglobin level of 10 g/dL (hematocrit 31%) and a normal coagulation profile (platelets 185000/mm³, prothrombin time 89%, international normalized ratio 1.06).

Following surgery, the patient was admitted to the intensive care unit (ICU) for close clinical and biological monitoring, in accordance with our institutional protocol of pre-emptive postoperative ICU admission for high-risk cesarean deliveries, particularly in the context of abruptio placentae with significant antepartum hemorrhage. Admission was further supported by the presence of persistent mild tachycardia in the immediate postoperative period.

Given the hemorrhagic context and as part of standard ICU admission practice at our institution, a right femoral central venous catheter (7 French, 20 cm, dual-lumen) was inserted using the conventional landmark-based technique by a senior resident in a single attempt, with the puncture site below the inguinal ligament and no clinically apparent distortion of anatomical landmarks at the time of insertion. The procedure appeared technically uneventful, with easy blood return on aspiration and no reported resistance or unusual sensation during guidewire or catheter advancement.

Postoperative laboratory results revealed a further decline in hemoglobin to 8.2 g/dL, prompting transfusion of packed red blood cells. A targeted etiological workup for abruptio placentae was conducted, including assessment for hypertensive disorders, thrombophilia screening, and infectious markers; no identifiable predisposing factor was found.

On postoperative day 1, the patient received two units of packed red blood cells. Follow-up laboratory testing paradoxically showed a further decline in hemoglobin to 7.8 g/dL, with a persistently normal coagulation profile. Clinical examination revealed no vaginal bleeding, no wound dehiscence, and a well-contracted uterus. Pelvic ultrasound, performed as part of the standard postpartum hemorrhage workup, revealed a small pelvic effusion.

On postoperative day 2, the patient developed worsening clinical and imaging findings, including increasing pelvic effusion on repeat ultrasound, mild abdominal distension, absent bowel sounds, and new-onset tachycardia (HR 110 bpm), without associated hypotension or need for vasopressor support. Serial hematological and coagulation values throughout the clinical course are summarized in Table 1.

**Table 1:** Serial laboratory values across the clinical course INR: international normalized ratio

Parameter	Admission	Postoperative	Day 1	Reference range
Hemoglobin (g/dL)	10	8.2	7.8	12.0-16.0
Hematocrit (%)	31	25	23	36-46
Platelets (×10³/mm³)	185	150	157	150-400
Prothrombin time (%)	89	75	71	70-100
INR	1.06	1.19	1.22	0.8-1.2

**Figure 1:**
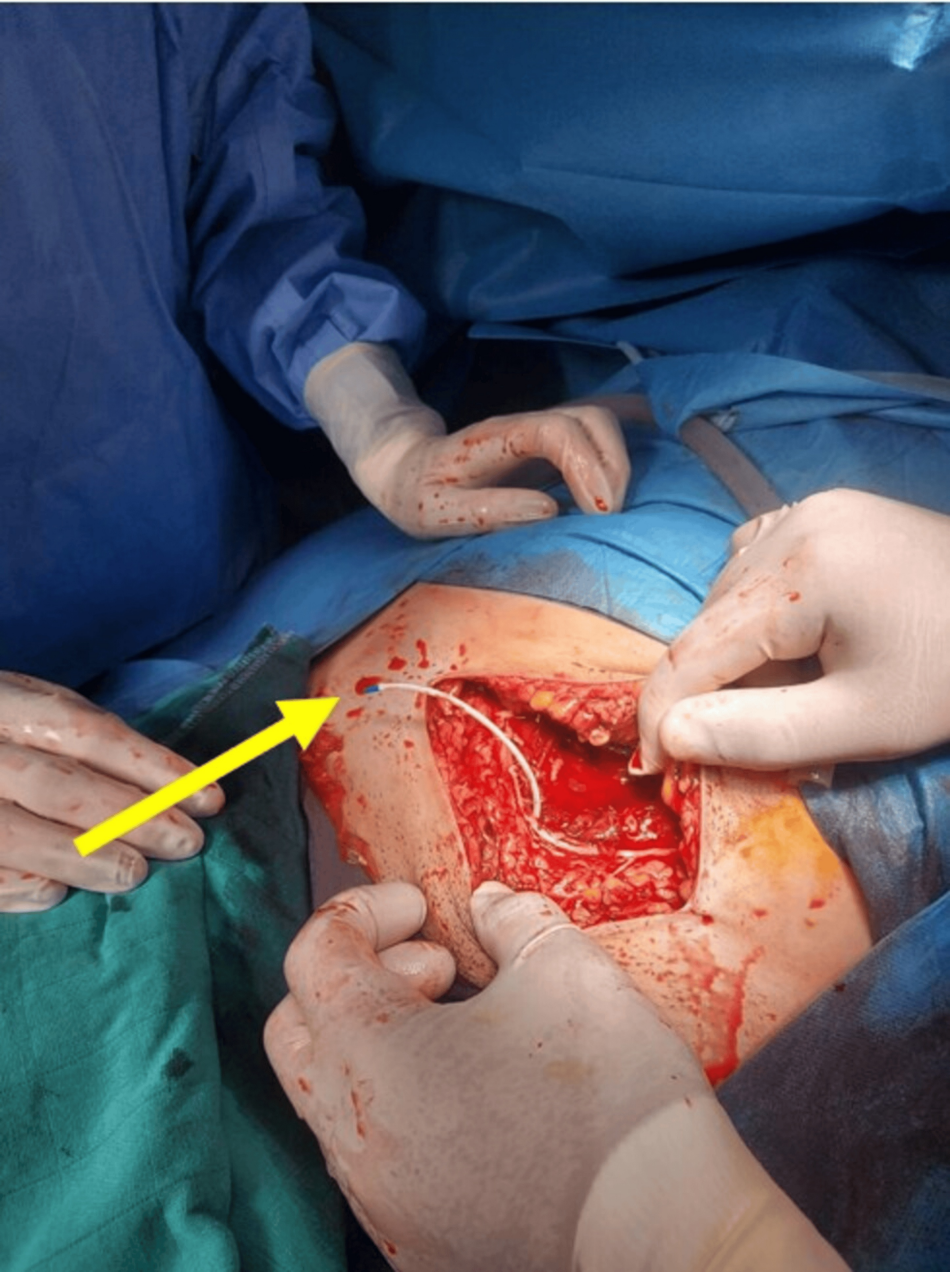
Intraoperative visualization of femoral catheter malposition. The yellow arrow indicates the catheter tip within the right pelvic peritoneal cavity

Given this clinical progression and suspected hemoperitoneum, a decision was made to return the patient to the operating room for surgical exploration. General anesthesia was induced uneventfully using an 18-gauge peripheral intravenous line. Surgical exploration revealed approximately 600 mL of hemoperitoneum, which was evacuated. Crucially, the tip of the right femoral central venous catheter was identified within the right pelvic peritoneal cavity (Figure 1).

The anesthesiology team performed catheter withdrawal under direct visual control, with only minimal residual bleeding at the pelvic entry site, which resolved with brief manual pressure and required no surgical hemostasis. The exact anatomical pathway by which the catheter reached the peritoneal cavity, whether through vascular perforation, injury during dilator passage, a fascial defect, or migration along a tissue plane, could not be determined, as complete vascular exploration was precluded by the unavailability of an on-site vascular surgery team. The mechanism of malposition, therefore, remains incompletely elucidated and constitutes a central point of discussion in this report.

The patient was readmitted to the ICU postoperatively with a favorable clinical evolution. She was transferred to the obstetrics ward on postoperative day 3 and subsequently discharged in stable condition.

## Discussion

Central venous catheterization (CVC) is one of the most frequently performed procedures in critical care and emergency medicine. The femoral vein represents one of the three main access sites alongside the internal jugular and subclavian veins, and is often selected in emergency settings because of its accessibility, the absence of pneumothorax risk, and the predictable anatomical course of the vessel.

Despite their widespread use, CVCs are associated with well-recognized complications, including mechanical complications at insertion (arterial puncture, hematoma, and pneumothorax), central line-associated bloodstream infections (CLABSI), and deep venous thrombosis (DVT). Overall complication rates for central venous access have been reported to reach approximately 15%, while the femoral approach is associated with a significantly higher incidence of infectious and thrombotic complications than subclavian access [3].

Beyond these established complications, rarer and more unusual events have been described, including catheter malposition into unexpected anatomical locations. Femoral catheters have been reported to migrate into the lumbar veins, the internal iliac vein, the peritoneal cavity, and, in patients with prior renal transplantation, into the transplant venous anastomosis [1,4-6]. Such malpositions carry potentially severe consequences, including intraperitoneal hemorrhage, abdominal compartment syndrome, and spinal cord injury [2,7].

To the best of our knowledge, this appears to be an exceptionally rare case of intraperitoneal malposition of a femoral venous catheter in a post-cesarean patient, presenting as an unexpected lack of response to blood transfusion and ultimately recognized during surgical exploration.

Understanding why the femoral route was selected in this clinical context is essential to contextualizing the complication. In obstetric emergencies complicated by hemorrhagic shock, femoral venous access is often selected over internal jugular or subclavian approaches for several clinically grounded reasons. First, the coagulopathy associated with major postpartum hemorrhage (up to 29%) may render non-compressible access sites such as the subclavian vein less desirable, as inadvertent arterial puncture in this context carries a higher risk of difficult-to-control bleeding [8]. Second, the femoral route offers rapid and reliable access without the risk of pneumothorax. Third, in resource-limited settings, familiarity with the landmark-based femoral technique and the absence of advanced monitoring equipment often make it the most pragmatic choice available.

The exact anatomical pathway by which the catheter reached the peritoneal cavity could not be fully elucidated intraoperatively, given the absence of an on-site vascular surgery team. However, four plausible mechanisms may be considered.

The most likely explanation is inadvertent mechanical injury to the vessel wall, leading to extravascular positioning of the catheter tip during advancement, specifically, perforation of the femoral vein wall, causing the catheter to track alongside the vessel rather than within its lumen. A second explanation is direct intraperitoneal placement from the outset, whereby the needle misses the vein entirely, follows a subcutaneous tract, and subsequently penetrates the peritoneum. A third explanation involves secondary malposition and migration after an initially correct placement, driven by patient movement, positioning, or local phlebitis [1]. A fourth possible mechanism is vascular injury during dilator passage in the Seldinger technique, prior to catheter advancement.

The clinical course of this case is instructive because the complication did not present with the dramatic signs one might expect from intraperitoneal hemorrhage. Instead, the dominant feature was a paradoxical and progressive decline in hemoglobin despite transfusion, a finding that, in the absence of external bleeding, coagulopathy, or surgical site complications, should raise suspicion of ongoing occult hemorrhage.

On postoperative day 1, hemoglobin fell from 8.2 to 7.8 g/dL despite transfusion of two units of packed red blood cells, a kinetic pattern not readily attributable to hemodilution or redistribution alone. No major coagulation abnormality was observed throughout, making consumptive coagulopathy an unlikely source of blood loss. The absence of vaginal bleeding, wound dehiscence, or uterine atony further narrowed the differential toward an intraperitoneal source. Diagnostic suspicion was driven by the convergence of an inadequate hemoglobin response to transfusion and progressive clinical and ultrasonographic findings, including increasing pelvic fluid accumulation, abdominal distension, and absence of bowel sounds.

A central question raised by this case is whether earlier confirmation of catheter placement could have prevented the delayed diagnosis and the need for surgical exploration.

Although bedside ultrasound was available at our institution, femoral venous cannulation in this case was performed using the conventional landmark-based technique without ultrasound guidance. This is notable in light of the 2020 American Society of Anesthesiologists Practice Guidelines for Central Venous Access, which support the use of real-time ultrasound for femoral venous access when feasible and emphasize that venous placement should not be confirmed solely by blood aspiration or nonpulsatile return [9].

Multiple confirmation modalities exist beyond simple aspiration. Point-of-care ultrasound can track the catheter trajectory in real time, detecting subcutaneous or extravascular advancement before full insertion. The FLUSH test, consisting of brisk saline injection during simultaneous cardiac ultrasound visualization, may provide a rapid bedside method to support intravascular tip confirmation. When malposition is strongly suspected, a CT scan of the abdomen and pelvis or emergency venography can provide definitive anatomical clarification.

The lessons from this case are clear: ultrasound guidance should be used systematically for femoral catheterization regardless of apparent technical ease, post-insertion confirmation should follow every insertion as a protocol rather than a selective practice, and blood return alone should not be considered sufficient proof of correct placement. The availability of a tool does not guarantee its use; this case strongly supports its routine integration into practice.

## Conclusions

This case illustrates an exceptionally rare but life-threatening complication of femoral venous catheterization, presenting not as overt hemorrhage but as a paradoxical decline in hemoglobin despite transfusion, a subtle pattern that ultimately guided the diagnosis. Standard insertion checks, including blood return on aspiration, failed to detect the malposition, reinforcing the principle that aspiration alone is insufficient confirmation of correct catheter placement.

Two lessons emerge. First, ultrasound guidance should be used whenever feasible for femoral catheterization in post-cesarean patients where pelvic anatomy may be altered, and paradoxical transfusion failure should prompt consideration of catheter-related complications in the differential diagnosis. Second, and more broadly, this case underscores the need for institutional protocolization of post-insertion catheter verification, even in resource-limited settings where bedside ultrasound is available but not systematically used. The gap between guideline recommendations and bedside practice remains a potentially preventable source of morbidity. Structured ultrasound training programs for residents and intensivists managing obstetric emergencies, combined with locally adapted confirmation protocols, represent a concrete and achievable step toward reducing this gap.
